# A Cognitive—Behavioral Intervention for Postpartum Anxiety and Depression: Individual Phone vs. Group Format

**DOI:** 10.3390/jcm10245952

**Published:** 2021-12-18

**Authors:** Meital Simhi, Orly Sarid, Heather Rowe, Jane Fisher, Julie Cwikel

**Affiliations:** 1 Spitzer Department of Social Work, Ben Gurion University of the Negev, POB 653, Beer Sheva 84105, Israel; orlysa@bgu.ac.il (O.S.); jcwikel@bgu.ac.il (J.C.); 2 The Center for Women’s Health Studies and Promotion, Ben Gurion University of the Negev, Beer Sheva 84105, Israel; 3 School of Public Health and Preventive Medicine, Monash University, Level 4, 553 St Kilda Road, Melbourne 3004, Australia; heather.rowe@monash.edu (H.R.); jane.fisher@monash.edu (J.F.)

**Keywords:** cognitive–behavioral intervention, mindfulness, perinatal mood and anxiety disorders (PMADs), group intervention, telephone consultation

## Abstract

Cognitive–behavioral interventions can effectively treat symptoms of perinatal mood and anxiety disorders (PMADs). We assessed the acceptability and effectiveness of a workbook-based intervention (What Am I Worried About? (WAWA)) comprising of cognitive–behavioral and mindfulness techniques and weekly professional guidance to address symptoms of depression, anxiety, and stress among postpartum mothers. We compared the efficacy of group versus individual telephone consultation using a pre-and post-test single group, open trial, research design in replication pilot study. A convenience sample of community-residing postpartum women (*n* = 34) chose between group intervention (*n* = 24) or individual phone consultation with a mental health professional (*n* = 10). Outcome measures were anxiety (GAD-7), depression, anxiety, and stress (DASS21), and postpartum depression (PPD-EPDS). After four weeks intervention, significant reductions were observed in postpartum depression, anxiety, and stress scales. Cohen’s *d* statistics showed medium effect sizes (0.35–0.56). A small but significantly larger change in PPD-EPDS and DASS stress scores was reported among participants who opted for the phone intervention compared to those in the group intervention. Most participants felt that the intervention was highly beneficial and would recommend it to other postpartum women. In conclusion, the WAWA intervention showed efficacy for reducing postpartum anxiety, distress, and depressive symptoms among postpartum women, with a slightly greater reduction in PPD-EPDS and stress symptoms found among those who opted for individual phone consultation. Definitive evaluation of the intervention requires a larger sample and a RCT research design with two treatment arms: telephone and group intervention.

## 1. Introduction

The prevalence of perinatal mood and anxiety disorders (PMAD, formerly called postpartum depression, PPD) in the general population worldwide is estimated at 10–20% [1,2]. Women who suffer from PMAD are vulnerable to emotional and physical risks, and symptoms of PMAD can have a negative effect on their infant’s development [3]. Cognitive–behavioral interventions (CBI) are an effective therapy for PMAD [2,4,5,6]. However, previous studies stressed the importance of choosing the suitable intervention format for postpartum women [7,8]. Thus, safe, effective, individually tailored, and non-pharmaceutical methods for the prevention and treatment of postpartum mental health problems are recommended [9].

What Am I Worried About (WAWA) is a workbook developed in Melbourne, Australia by Rowe and Fisher to provide a low-cost, accessible method for treating postpartum women who experience mild to moderate symptoms of PMAD [4]. WAWA combines cognitive behavior and mindfulness techniques and can be delivered in person to individuals or within a group meeting, by telephone or ZOOM consultations with the guidance of a mental health professional

Existing evidence suggests that treatment offered in a group setting can be effective for reducing postpartum depressive symptoms among women [8,9,10,11,12,13]. A possible advantage of a group setting for managing PMAD symptoms is that it capitalizes on the interactions between participants, thereby normalizing feelings that may otherwise be disturbing, and it mitigates the sense that they are alone in managing difficulties [14]. Previous studies have indicated that group interventions can provide opportunities for building supportive contacts and bolstering network connections, thus reducing the isolation felt by many new mothers. The group format also offers the opportunity to learn by modeling others who are successfully managing similar challenges [13]. However, formal group meetings can be a challenge to mothers caring for an infant [13,15,16]. In this case, mothers might prefer individual counseling via telephone, ZOOM, or Skype. Studies that evaluated the effect of CBI delivered via telephone for postpartum mothers have found that this format is feasible and effective in reducing depressive symptoms [14,15,16,17,18]. However, possible challenges in this intervention format include interruptions during sessions, technical problems with phone or internet connections, difficulty understanding important concepts when relying only on auditory cues, and lack of physical proximity that can limit the establishment of a therapeutic alliance [19]. Recent findings from a community study showed that postpartum women preferred receiving treatment for postpartum depression by a health professional in a group format in contrast to an individual phone consultation [8]. Thus, we hypothesized that more mothers would prefer to participate via a group intervention compared to individual phone consultation.

Findings from a recent meta-analysis showed that no study to date had compared between group and individual-delivered CBI for PMAD [20]. This was the rationale for the current study. The aims of the current study were (1) to assess the feasibility and effectiveness of the WAWA intervention with professional guidance among postpartum women, using standardized instruments to evaluate symptoms of depression, anxiety, and stress before and after the intervention, and (2) to examine whether phone or individual format produced better results on symptoms of depression, anxiety, and stress.

## 2. Materials and Methods

We conducted a replication pilot study, using a pre-and post-test single group research open trial design. In the current study, we translated the original WAWA workbook into Hebrew and then checked the translation by back-translating it into English, which was checked for accuracy by a fluent bilingual team member (J.C.). The original WAWA developers (H.W. and J.F.) acted as consultants for the project in order to ensure consistency of intervention style.

Inclusion criteria were women over 18 years, who had given birth in the last 3–6 months, and could read and write fluently in Hebrew. Exclusion criteria were any known diagnosed psychiatric conditions. The protocol was approved by the Spitzer Department of Social Work, Ben Gurion University of the Negev, Ethics Committee.

### 2.1. Procedure

**Figure 1:**
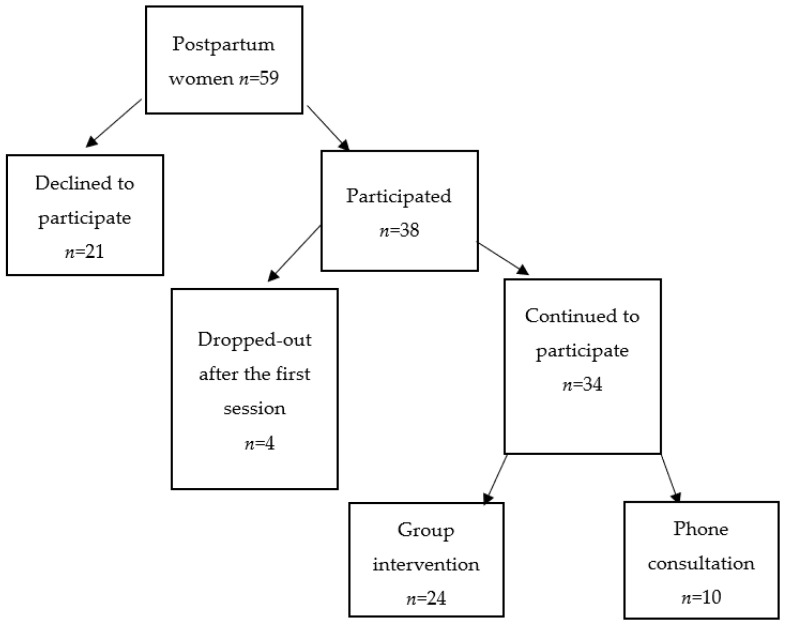
Participant flow for treatment: Group versus individual telephone consultation.

A convenience sample was recruited through social media. A flyer advertising the group was posted on Facebook, and after some women responded, we used snowball-sampling techniques to recruit additional participants. Postpartum mothers were invited to attend an initial meeting if they felt overwhelmed and stressed in the months after delivery. In the initial meeting, the study aims were presented, and general information on anxiety, depression and other common reactions was given by the health professional, who was an experienced clinical social worker (M.S.). Postpartum women who expressed interest and willingness to participants in the study signed a consent form and filled in the baseline questionnaire (Time 1-T1). Following the open trial protocol, participants were offered the option of group meetings or individual telephone counseling. Group meetings were held in the community once a week for four weeks. In a similar manner, individual consultation was provided once a week for four weeks via telephone. The group and the individual counseling were delivered by the same mental health professional (M.S.) and supervised by J.C. and O.S. Participants received no remuneration for participation in the study. The T2 questionnaires were completed at the last meeting or by email for participants in the individual phone format. The flow of the sample into intervention groups is presented in Figure 1.

### 2.2. Sample

Fifty-nine women met the inclusion criteria and were invited to participate. Out of these, thirty-eight women attended the initial informational meeting, signed an informed consent form, and agreed to participate (64% response rate). Four women dropped out after the first intervention session, leaving 34 women who completed the intervention with pre and post data (89% completion rate). A similar sample size was used in several studies that examined interventions for postpartum depressive women [11,21,22,23].

The great majority of the participants were married, Israeli born, with a mean age of 30.79 (SD = 3.93). Participants had on average 14.88 years of education (SD = 2.04). The majority worked in paid employment prior to the current birth, and about half stated that they had no economic difficulties (see Table 1). On average, participants had four children (SD = 2.12), including the index infant who was between three and six months at the start of the intervention.

### 2.3. Research Instruments

T1 and T2 questionnaires included standardized measures and study-specific questions:

GAD-7-the General Anxiety Questionnaire [24]. This is a 7-item self-report measure of anxiety symptoms that is commonly used in community studies. Higher scores indicate more severe symptoms. Cronbach’s alpha in T1 and T2 was 0.77 and 0.79 respectively.

DASS 21-Depression Anxiety Stress Scale [25,26]. This is a 21-item self-report measure of current depression, anxiety, and stress symptoms, comprising three sub-scales with seven items each. Higher scores indicate more severe symptoms. Cronbach’s alpha in T1 and T2, D = 0.82, 0.74; A = 0.70, 0.40; S = 0.91, 0.85, respectively.

PPD-EPDS-Edinburgh Postnatal Depression Scale [27]. This is a 10-item self-report scale commonly used to detect clinical indicators of postpartum depression. Cronbach’s alpha in T1 and T2 was 0.83 and 0.76, respectively.

Sociodemographic data were collected in T1: family status, education, age, religiosity, country of birth, economic difficulties, employment status before delivery, employment status of partner, and number of children.

At T2, women answered a structured questionnaire measuring ways of thinking related to baby care, parenting, and cognitive function, before and following the intervention: Participants were asked two questions: “In what areas did you feel anxious or distressed at the start of the intervention?”; “In what areas did you feel less anxious or distressed after the intervention?” These questions were not asked at T1, given that the work of the WAWA intervention included recognizing these specific maladaptive thought patterns. Thus, the T2 evaluation is a retrospective view of how their thinking and reacting might have changed during and after the intervention. More than one answer could be reported: e.g., dealing with my baby’s crying, feeding my baby, dealing with inconsistent information, my baby’s safety and health, leaving my baby in the care of others, comparing myself to others, comparing myself to who I was before the birth, and leaving home with or without the baby.

Cognitive patterns that were addressed in the therapeutic tasks of WAWA were assessed with two multiple-choice questions: “What kinds of thoughts did you have at the start of the intervention?”, and “What kinds of thoughts did you feel that you were able to change through the intervention? “. More than one answer could be reported, including changing to more flexible thought patterns, less catastrophizing and overgeneralizing, fewer “should” and “must” statements, less personalization, decreased jumping to conclusions, and less mind-reading.

The T2 questionnaire also included three questions to measure participants’ practice frequency of the recommended coping techniques, each on a five-point Likert scale ranging from ‘not at all’ to’ more than once a day’, including relaxation exercises, thought reframing, and mindfulness.

Three questions evaluated the intervention overall, each on a five-point Likert scale ranging from very negative to very positive. These included (1) a general question regarding the quality of the WAWA intervention, (2) whether working using the workbook had met their needs, and (3) to what extent they would recommend this intervention to other women managing mental health symptoms after childbirth.

### 2.4. The WAWA Protocol

The intervention was delivered using a self-help workbook designed to address common anxieties that are experienced by new mothers and ameliorate symptoms of depression by practicing cognitive behavioral and mindfulness techniques [4]. A CBI mental health professional teaches and practices with the women as they progress through the workbook.

An overview of the content of the four sessions is presented in Table 2.

### 2.5. Statistical Methods

Using SPSS version 25 (IBM, Armonk, NY, USA), the data were cleaned and checked for outliers. Standard descriptive measures (mean, standard deviation, and mode) were calculated for the dependent and demographic variables. In order to ensure the homogeneity of the sample, in the first stage of the analyses, we used one-way ANOVA analyses between all dependent variables by the degree of religiosity, which is an important potential mediating variable. There were no statistically significant differences in outcome measures between the religious groups (secular, traditional, religious, or ultra-Orthodox). Thus, religiosity was not included in the remaining analyses as a covariate. We tested differences between the T1 and T2 values of the dependent variables both for the whole sample and between the two groups using independent sample t-tests. Cohen’s d effect sizes for all dependent variables were calculated for paired samples. We compared the effect sizes by intervention format (group or individual phone). Change in dependent variables from T1 to T2 (Deltas) were also calculated. We calculated the change in issues of infant care that aroused anxiety and maladaptive thought patterns that the participants attributed to their participation in the WAWA intervention. Then, we calculated chi-squares to test whether the changes reported were more common about the group or telephone intervention participants.

## 3. Results

The group format was chosen by 71%, thus confirming the hypothesized format preference of group over individual phone consultation.

All psychological measures: symptoms of postpartum depression (EPDS), anxiety (GAD), DASS (depression, anxiety, and stress) showed significant reductions following the intervention (see Table 3). Cohen’s *d* effect sizes analyses for paired samples showed that they were in the medium effect size range (0.35–0.56).

The mean EPDS and DASS scores of the respondents who chose individual phone versus group settings did not differ significantly from each other at baseline (t (32) = 1.863, *NS;* t (32) = 1.629, *NS,* respectively). However, statistically significant differences in deltas for EPDS and DASS stress scores were observed (see Table 4) between participants in individual phone consultation versus group sessions. Participants who worked individually by phone reported lower PPD-EPDS (M (SD) = 4.8 (5.09)) and DASS stress scores (M (SD) = 3.33 (4.44)) than those who chose the group format. No differences were observed on GAD anxiety scores or DASS depression or anxiety scores.

The concerns reported by participants as arousing the most anxiety prior to the intervention are presented in Table 5. Following the intervention, participants for whom it was a concern in T1 reported improvements in the stress associated with care and security of their infant; comparing herself to others; comparing herself to her prenatal self; caring for her crying baby, feeding her baby, and dealing with inconsistent information. The maladaptive cognitive patterns reported by participants prior to the intervention were also analyzed. Following the intervention, participants for whom it was a concern in T1 learned to decrease their tendency to catastrophize or overgeneralize situations and reduced their tendency to believe that they could read other people’s minds. About two-thirds of those who were concerned about leaving their infant felt more relaxed about that, and over 60% of those who used to overstate dangers were able to reduce this tendency. About two-fifths reduced their “should” and “must“ statements and learned not to jump to conclusions, and one-third of the participants learned to not take everything personally (see Table 5).

In response to questions summarizing their experiences with the intervention (not shown in Table 5), 73% felt that the intervention was highly beneficial, and 27% felt that it was somewhat beneficial. About three-quarters (73%) felt that the methods learned and practiced were highly useful, whereas less than one-fifth felt that their needs were not answered to some extent, and only 9% felt they did not benefit at all. The usefulness of the method was reflected in the high percentage who would recommend the intervention to other women (97%).

No significant differences were found in chi-square analyses between those who received the intervention by phone or in the group, in terms of their cognitive, behavioral, or mindfulness practice (χ^2^ (4) = 5.91, *p* > 0.05; χ^2^ (4) = 6.38, *p* > 0.05; χ^2^ (4) = 3.43, *p* > 0.05), respectively.

Regarding cognitive techniques (not shown in table), 24% reported that they reframed their thoughts more than once a day, 21% did so once a day, 37% reported that they reframed their thoughts a few times a week, 12% reported of using the technique once a week, and 6% did not use the technique.

Only 12% practiced behavior techniques such as relaxation exercises daily, 44% reported doing so once or a few times a week, and 44% reported that they did not practice behavior techniques at all.

Mindfulness was sometimes practiced: 16% reported that they practiced mindfulness exercises daily, 53% reported doing so once to a few times per week, and 31% reported that they did not practice mindfulness exercises.

## 4. Discussion

This study assessed the acceptability and effectiveness of a cognitive–behavioral and mindfulness intervention based on the WAWA workbook designed to relieve common symptoms of mild to moderate anxiety and depression following childbirth. The intervention was delivered in either an individual phone consultation or group format according to an open trial format, which allowed women to choose the intervention style that best fit their personal preferences and circumstances.

Our findings showed that following the intervention, there was a significant reduction in depression, anxiety, and stress symptoms in both intervention formats. These findings are consistent with previous studies showing that CBIs are a safe and effective therapy for PMAD [2,4,5,6], which can be delivered effectively in either group or individual intervention format.

The mean levels of depressive symptoms of the individual phone and groups did not differ significantly at baseline; thus, we can rule out that the more stressed or depressed participants preferred one modality over the other. Following the intervention, no significant differences were found between those who received the intervention by phone or in the group format in the indicators of anxiety and depression (as measured by DASS). A significantly greater change was apparent in PPD-EPDS and stress among those who worked individually by phone compared to the group intervention participants. Studies that have evaluated the effect of CBI delivered via telephone for new mothers have found that this format was feasible and effective in reducing depressive symptoms during the postpartum period, yet they did not compare the intervention with another treatment-delivery modality [15,16,17]. In addition, a recent study found that both group and individual formats for CBT for PMAD symptoms were equally effective [20]. Our findings support this conclusion but suggest that on some measures, the individual format was associated with results that were more significant. We suggest that both methods can be effective and therefore should be offered to women at risk of PMAD, allowing them to choose the format that suits their needs. Future studies should replicate this intervention in other cultures and populations, including among women who are single and/or belong to ethnic minority and immigrant groups.

We also found that almost all mothers in our study felt the intervention met their needs. The majority felt that WAWA was beneficial and would recommend this type of intervention to other mothers. The findings are consistent with the original WAWA program evaluation [4] in which participants chose whether to continue attending the group program or to go over to working individually by phone, and this choice seemed to encourage engagement in the intervention. The present study also showed that being flexible regarding the mode of intervention delivery allowed women to access treatment within their own life constraints while managing infant care and encouraged participation.

This replication study included mostly religious women who usually do not participate in postnatal mental health interventions and are considered a high-risk population [28]. By tailoring the WAWA intervention to their needs by offering either individual phone or small group interventions, we maintained a high level of retention in the study once mothers decided to participate.

The primary limitations of this study were the small sample size and the absence of a control group with an RCT design that could effectively rule out alternative explanations for the results such as regression toward the mean among a self-selected sample. Thus, we highly recommend replication with a larger sample and use of the RCT research design. In a future RTC study, a statistical power analysis is recommended to calculate the desired sample size, which is something that we were not able to do given the absence of a control group and the pre and post-test research design. Another limitation lies in the dropout from the intervention, particularly after the initial informational meeting that may raise questions about the generalizability of the findings. Perhaps the twenty- one women who dropped out after attending the initial informational meeting or the four who dropped out after the first intervention meeting did not see themselves benefitting from the intervention or they had instrumental barriers that prevented them from participating in the program.

The results of the present study can inform the protocol design for future WAWA evaluations. Future studies should also include other “at-risk” populations in order to generalize the benefits identified in this study. An additional limitation was that the interventions were delivered by the same mental health practitioner (M.S.). The use of the same mental health professional across mode of delivery helps limit potential bias that may have arisen from between-therapist differences; however, to increase the generalizability of the findings, it is desirable to use multiple therapists to test the fidelity of the WAWA.

## 5. Conclusions

The WAWA intervention is a safe, feasible, acceptable, and effective method for addressing mild to moderate post-partum anxiety, stress, and depression symptoms among women in the community. Offering women a choice between group, individual, telephone, or other online formats seemed to increase engagement. Online or telephone formats may reduce stigma and have the benefit of maintaining social distancing, isolation, and quarantine restrictions during the COVID-19 pandemic. The successful implementation of a mental health intervention in an e-therapy format suggests an ongoing niche for telehealth [29]. Previous research suggests that internet-delivered treatment is effective for women in the postnatal period [30,31]. Therefore, further studies should also examine the effectiveness of the WAWA intervention using digital platforms such as ZOOM. An online platform may overcome COVID-19 quarantine restrictions as well as regular face-to-face treatment barriers such as mobility, stigma, and childcare concerns.

## Figures and Tables

**Table 1:** Sociodemographic characteristics of the whole sample and by intervention format.

Variables	*n* = 34 (%)	Individual *n* (%)	Group *n* (%)
Family status (*n* = 32)			
Married	31 (97)	9 (28)	22 (69)
Divorced	1 (3)	0	1 (3)
Children (*n* = 33)			
First child	6 (18)	2 (6)	4 (12)
<Second child	27 (82)	8 (24)	19 (58)
Religious status (*n* = 33)			
Secular	1 (3)	0	1 (3)
Traditional	2 (6)	1 (3)	1 (3)
Religious	26 (79)	6 (18)	20 (61)
Ultra-Orthodox	4 (12)	2 (6)	2 (6)
Country of origin (*n* = 34)			
Immigrant	2 (6)	0	2 (6)
Native born	32 (94)	10 (29)	22 (65)
Employed (*n* = 34)			
Yes	31 (91)	9	22
No	3 (9)	1	2
Spouse employment (*n* = 34)			
Unemployed	2 (6)	1 (3)	1 (3)
Employed	32 (94)	9 (26)	23 (68)
Economic status (*n* = 34)			
Some difficulty	15 (45)	7 (21)	8 (24)
No difficulty	19 (55)	3 (8)	16 (47)

**Table 2:** The WAWA protocol, by weekly session [4].

Session Number	Topics Discussed	Homework Assigned
Initial introductory meeting	Introduction to the intervention principles presented in the workbook. Participants signing informed consent forms received a personal workbook.	
Session 1	Background information on how symptoms of anxiety, depression, and distress present in the post-partum period. The links between thoughts, feelings, and behaviors are presented.	Identify thoughts that preceded distressing feelings and behaviors and write them down.
Session 2	Participants were taught to discern maladaptive thought patterns such as dichotomized thinking (e.g., good–bad), catastrophizing, ‘should or must’ self-talk, etc. Participants learned to reframe maladaptive thoughts, in general, and in relation to mothering their infant, into more realistic, flexible patterns, and were encouraged to remember when things have gone well. Relaxation techniques to reduce stress were taught.	Identify and write down thoughts concerning infant’s feeding, crying, and other disturbing situations. Reframe thoughts into more flexible and adaptive patterns. Practice breathing and relaxation.
Session 3	Using examples from daily life, mothers learned to manage inconsistent information regarding infant health and safety and deal with feelings of anxiety when they leave the infant with another person. Mindfulness and breathing skills were taught and practiced.	Identification of thoughts regarding infant behaviors and mother’s responses that were learned in the session. Reframing thoughts and assessing the feelings and behaviors derived from more adaptive ways of thinking. Practice mindfulness and breathing.
Session 4	Mothers shared their personal experiences. Cognitive patterns of comparing oneself with others and how ‘I used to be’ were discussed and addressed with cognitive restructuring techniques. Skills attained over the intervention were highlighted and reinforced.	Mothers were encouraged to keep practicing relaxation techniques and to use their more adaptive modes of thinking and behaving after the intervention.

**Table 3:** Before (T1) and after (T2) comparisons of dependent variables (EPDS, GAD, DASS) (*n* = 34).

Dependent Variables	Mean	(SD)	t (df = 33)	Cohen’s *d* Effect Size
EPDST1	5.88	(4.43)	3.28 **	0.56
EPDST2	3.67	(3.06)		
GADT1	5.97	(3.66)	2.53 **	0.45
GADT2	4.34	(3.18)		
DASS Stress T1	4.94	(4.05)	2.204 *	0.40
DASS Stress T2	3.52	(2.70)		
DASS Anxiety T1	1.44	(2.03)	2.050 *	0.35
DASS Anxiety T2	0.79	(1.20)		
DASS Depression T1	3.06	(2.75)	2.651 **	0.45
DASS Depression T2	1.88	(1.99)		

* *p* < 0.05; **, *p* < 0.01.

**Table 4:** Mean delta differences (T1–T2) by intervention type (telephone, group) (*n* = 34).

Dependent Variables	Format of Intervention	*n*	Mean	(SD)	t (df = 33)
Delta EPDS	telephone	10	4.80	(5.09)	2.72 *
	group	24	1.12	(2.78)	
Delta GAD	telephone	10	0.80	(5.37)	−0.86 (ns)
	group	22	2.00	(2.58)	
Delta DASS stress	telephone	9	3.33	(4.44)	2.00 *
	group	22	0.63	(2.93)	
Delta DASS anxiety	telephone	10	1.20	(2.14)	1.03 (ns)
	group	24	0.41	(1.69)	
Delta DASS depression	telephone	10	1.30	(3.33)	0.15 (ns)
	group	24	1.12	(2.29)	

* *p* < 0.05.

**Table 5:** Percentages of responses on issues that aroused anxiety before (T1) and after the intervention (T2), *n* = 34.

	T1 *n* (% of the Sample)	T2 *n* (% Reduction among Those with Concern at T1 *, % of the Sample)
My baby’s safety and health	18 (55)	15 (17,45)
Comparing myself to others	16 (49)	10 (37,30)
Comparing myself to who I was before	15 (45)	12 (20,36)
Leaving my baby to be cared for by others	12 (36)	4 (67,12)
Leaving home/going out with baby Feeding my baby	8 (24) 7 (21)	5(37,15) 5 (29,15)
Dealing with inconsistent information	6 (18)	4 (33,12)
Maladaptive Cognitive Patterns		
“Should” and “must” statements	26 (76)	16 (39,47)
Catastrophizing	17 (50)	16 (6,47)
Overgeneralizing	17 (50)	15 (12,44)
Jumping to conclusions	12 (35)	7 (42,21)
Personalization	10 (29)	7 (30,21)
Mind reading	10 (29)	9 (10,26)
Overstating the danger	8 (24)	3 (62,8)
Underestimating one’s coping ability	8 (24)	6(25,18)
Filtering	7 (21)	6 (14,18)
Dichotomous thinking	7 (21)	5 (29,15)

* Reduction was calculated by 100% of those with concern at T2 among those who reported this concern at T1.

## Data Availability

The datasets used and/or analyzed during the current study are available from the corresponding author on reasonable request.
